# Investigation on explainable machine learning models to predict chronic kidney diseases

**DOI:** 10.1038/s41598-024-54375-4

**Published:** 2024-02-14

**Authors:** Samit Kumar Ghosh, Ahsan H. Khandoker

**Affiliations:** Department of Biomedical Engineering & Biotechnology, Khalifa University, Abu Dhabi, United Arab Emirates

**Keywords:** Kidney, Kidney diseases, Renal replacement therapy, Kidney diseases

## Abstract

Chronic kidney disease (CKD) is a major worldwide health problem, affecting a large proportion of the world’s population and leading to higher morbidity and death rates. The early stages of CKD sometimes present without visible symptoms, causing patients to be unaware. Early detection and treatments are critical in reducing complications and improving the overall quality of life for people afflicted. In this work, we investigate the use of an explainable artificial intelligence (XAI)-based strategy, leveraging clinical characteristics, to predict CKD. This study collected clinical data from 491 patients, comprising 56 with CKD and 435 without CKD, encompassing clinical, laboratory, and demographic variables. To develop the predictive model, five machine learning (ML) methods, namely logistic regression (LR), random forest (RF), decision tree (DT), Naïve Bayes (NB), and extreme gradient boosting (XGBoost), were employed. The optimal model was selected based on accuracy and area under the curve (AUC). Additionally, the SHAP (SHapley Additive exPlanations) and LIME (Local Interpretable Model-agnostic Explanations) algorithms were utilized to demonstrate the influence of the features on the optimal model. Among the five models developed, the XGBoost model achieved the best performance with an AUC of 0.9689 and an accuracy of 93.29%. The analysis of feature importance revealed that creatinine, glycosylated hemoglobin type A1C (HgbA1C), and age were the three most influential features in the XGBoost model. The SHAP force analysis further illustrated the model’s visualization of individualized CKD predictions. For further insights into individual predictions, we also utilized the LIME algorithm. This study presents an interpretable ML-based approach for the early prediction of CKD. The SHAP and LIME methods enhance the interpretability of ML models and help clinicians better understand the rationale behind the predicted outcomes more effectively.

## Introduction

Chronic kidney disease (CKD) is a progressive and often debilitating medical condition characterized by the gradual loss of kidney function over time. It is defined by a glomerular filtration rate (GFR) under $60\,\text {mL}/\text {min}/1.73\,\text {m}^{2}$ for over 3 months or the presence of kidney damage indicators^1,2^. Common causes of CKD include diabetes, high blood pressure, glomerulonephritis, and other related conditions^3^. It affects millions globally and creates a significant healthcare burden due to its high prevalence, costly treatments, and potentially life-threatening complications^4^. Early detection and identification of critical risk factors are crucial to managing CKD effectively and improving patient outcomes. Traditional diagnostic approaches are ineffective in properly predicting the progression of CKD and identifying the most significant factors contributing to its development. However, recent advancements in computational intelligence and machine learning (ML) techniques have shown great promise in terms of revolutionizing CKD diagnosis and variable importance analysis^5–8^. This introduction investigates the relationship between computational intelligence and CKD diagnosis, emphasizing the use of ML techniques to predict and diagnose the disease at early stages. It also explores the idea of variable importance, highlighting its significance in understanding the underlying factors driving CKD progression and the development of tailored treatment strategies.

Over recent years, various studies have focused on the effective and precise diagnosis of CKD patients. Diverse classifiers have been employed to establish classification models to categorize clinical CKD data, as evidenced by the work of Jena et al.^9^. The investigation carried out by Ahmad et al.^10^ utilized supervised ML techniques, including neural networks (NN), logistic regression (LR), and support vector machines (SVM), to achieve this objective. A unified approach combining classification and association rule mining techniques has been employed to design a system for predicting and diagnosing CKD and its underlying causes. Among the diverse algorithms explored on medical data are random forest (RF), *k*-nearest neighbors (KNN), decision trees (DT), Naïve Bayes (NB), LR, and SVM. Moreover, Alloghani et al.^11^ documented using the Apriori algorithm to choose attributes from the CKD dataset. This approach aimed to extract robust rules using the lift matrix.

In recent decades, there has been a notable increase in studies effectively diagnosing CKD patients with high accuracy. Wibawa et al.^12^ employed a combination of KNN and AdaBoost, utilizing the correlation-based feature selection (CFS) technique to select 17 attributes out of 24, resulting in an impressive accuracy of 98.1%. Polat et al.^13^ achieved a remarkable 98.5% accuracy using a SVM classifier with a filter subset evaluator method for feature selection, identifying 13 relevant features out of 24. Taznin et al.^14^ conducted research on a CKD dataset, achieving a notable 99% accuracy using the DT algorithm with only 15 attributes out of 24 features. Similarly, Amirgaliyev et al.^15^ attained a notable accuracy of approximately 94.60% using an SVM classifier with all 24 attributes. Yildirim et al.^16^ employed multilayer perceptron (MLP) and a sampling technique, achieving a noteworthy F1 score of 99.8%. On the same dataset, Salekin et al.^17^ achieved a remarkable 99.3% F1 score with a RF classifier, demonstrating similar results (99% F1 score) with only ten relevant predictive attributes. Rubini et al.^18^ utilized the fruit fly optimization algorithm (FFOA) for feature selection, identifying 11 relevant attributes out of 25 and achieving an accuracy of 99.08% using multi-kernel SVM (MKSVM) as the classifier. Emon et al.^19^ tested several ML algorithms and found that RFC exhibited the highest accuracy at 99%. Gupta achieved 99.24% accuracy in the same dataset using LR^20^. Manonmani et al.^21^ applied the improved teacher-learner-based optimization (ITLBO) algorithm as a feature selection technique, obtaining 16 features out of 24. They achieved an accuracy of 99.25% using the convolutional neural network (CNN) classification algorithm. Gunarathne et al.^22^ reduced attributes from 24 to 14 using the multiclass decision forest (MDF) method, resulting in 99.1% accuracy. Avci et al.^23^ classified CKD, attaining 99% accuracy with the J48 classifier. Aswathy et al.^24^ introduced a flower pollination algorithm (FPA)-based deep neural network (DNN), an innovative CKD diagnosis model leveraging Internet of Things (IoT) and cloud technologies. Demonstrating superior performance in sensitivity, specificity, accuracy, F-score, and kappa, the model utilized the FPA and oppositional crow search (OCS) for feature selection, suggesting future advancements in CKD diagnosis. Suliman et al.^25^ proposed an ensemble of deep learning-based clinical decision support systems (EDL-CDSS) for CKD diagnosis in an IoT-enabled cloud environment. The approach, incorporating data gathering, preprocessing, Adaptive Synthetic (ADASYN) outlier detection, and an ensemble of deep learning models with quasi-oppositional butterfly optimization algorithm (QOBOA) hyperparameter tuning, showed superiority over existing methods in simulations. In 2018, Shamsi et al. employed a statistical methodology, specifically the multivariable Cox’s proportional hazards analysis, to evaluate high-risk cardiovascular disease (CVD) patients. The purpose of this study was to determine independent risk factors, such as age, history of coronary heart disease (CHD), diabetes mellitus (DM), and smoking, for the development of CKD stages 3–5^26^. Davide et al. developed ML techniques for predicting CKD stages 3–5 using the same CKD dataset. Additionally, they conducted a feature ranking analysis to understand the most crucial clinical factors^27^.

The reviews above indicate that numerous studies have been conducted on predicting CKD using ML techniques. Various parameters, such as dataset size, dataset quality, and the timing of dataset collection, play significant roles in enhancing model performance. However, many of these earlier models faced challenges in clinical implementation because of their lack of interpretability and the “black-box” nature of their algorithms^28,29^. Understanding this “black box” is essential since it helps physicians understand the inner workings of ML models^30^. Explainable artificial intelligence (XAI) was developed to address this issue and explain how machine learning (ML) makes decisions. Explainable ML is concerned with increasing the transparency and credibility of “black box” model decision-making. Among the prominent XAI techniques are the SHapley Additive exPlanation (SHAP) and Local Interpretable Model-agnostic Explanations (LIME)^28,31^. These techniques have demonstrated their effectiveness in providing valuable insights into machine learning models, particularly those related to CKD prediction. However, there seems to be a lack of studies on the dependability and robustness of these explanatory approaches when predicting outcomes for CKD.

The novelties of the present study with respect to the state-of-the-art are: (1) it focuses on predicting kidney disease by employing a variety of ML algorithms such as LR, RF, DT, and NB; (2) the research evaluates the proposed method against XGBoost, a leading-edge algorithm, for predicting overall survival outcomes in CKD patients; (3) the study elucidates the predictions of the XGBoost model through the application of SHAP and LIME methods; (4) it demonstrates how these interpretable models can contribute to the medical field by forecasting patients’ survival probabilities; (5) the study underscores the significance of personalized risk stratification through the developed model, highlighting its potential to assist clinicians in tailoring treatment intensity based on the unique prognostic profiles of individual patients.

## Materials and method

### Data source and description

**Figure 1:**
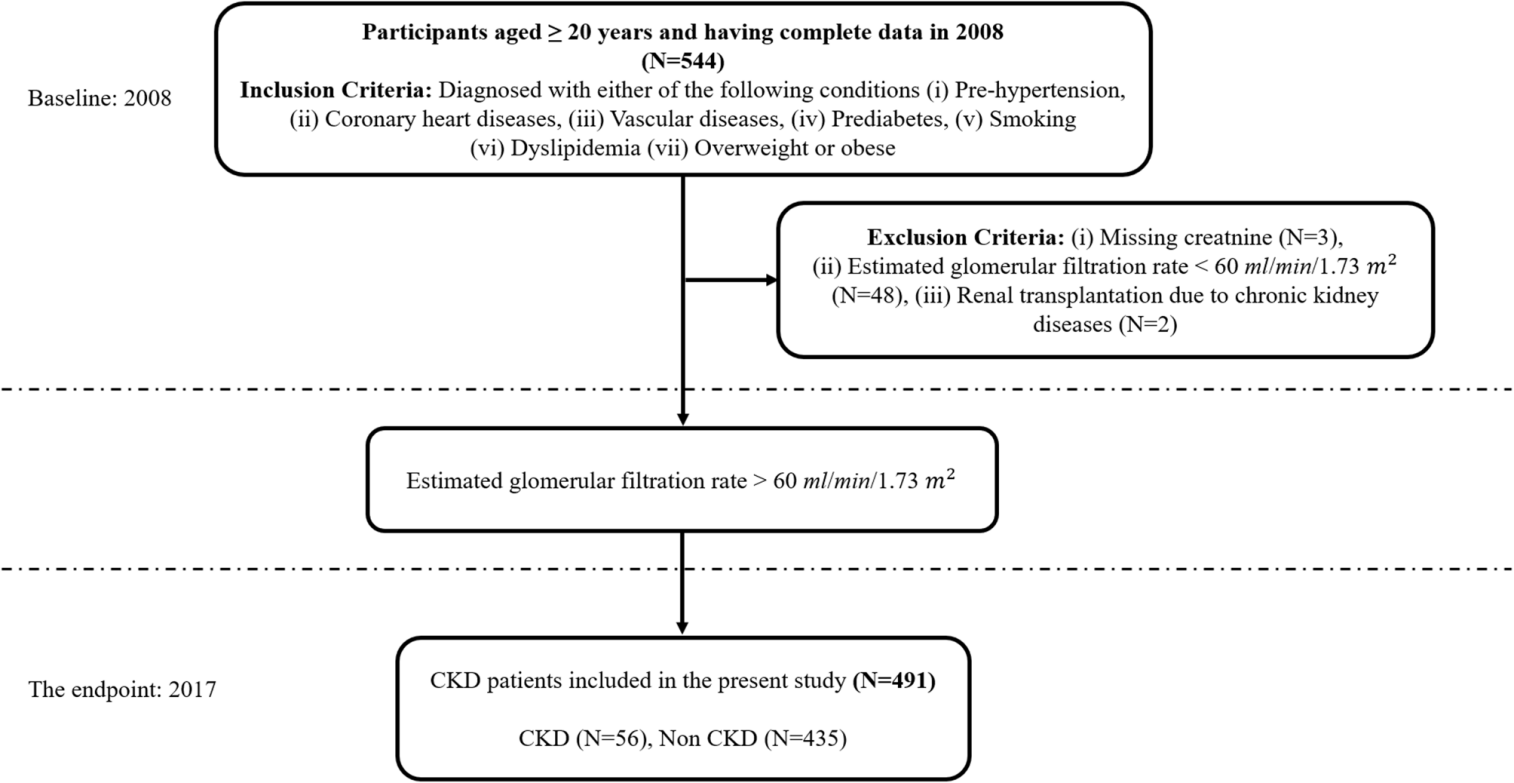
Flow diagram of the cohort study.

The dataset used in this work was obtained from 544 patients admitted to Tawan Hospital in Al-Ain City, Abudhabi, United Arab Emirates (UAE). The data were collected between January 2008 and December 2008^26^. The Tawam Hospital and UAE University research ethics board approved the study protocol (Application No. IRR536/17). Informed consent was not required since patient data and information were anonymized and de-identified prior to analysis. All the experiments were performed in accordance with the approved guidelines and complied with the Declaration of Helsinki. The data details are publicly available at the following link: https://figshare.com/articles/dataset/6711155?file=12242270. The flow diagram of the cohort study, which includes the inclusion and exclusion criteria, is depicted in Fig. 1.

The dataset comprises demographic, biochemical, and clinical data pertaining to patients with CKD. The distinctive characteristics include gender, age, history of coronary heart disease (CHD), history of diabetes, history of vascular diseases, history of smoking, history of hypertension (HTN), history of dyslipidemia (DLD), history of obesity, medications for dyslipidemia (DLD), medications for diabetes (DM), ACEIARB, cholesterol levels, triglyceride levels, glycosylated hemoglobin type A1C (HgbA1C), creatinine levels, estimated glomerular filtration rate (eGFR), systolic blood pressure (SBP), diastolic blood pressure (DBP), body mass index (BMI), and follow-up duration.

The categorical features include the patient’s gender. Additionally, personal history factors are considered, such as diabetes history, CHD history, vascular disease history, smoking history, HTN history, DLD history, and obesity history. Disease-specific medications, namely DLD medications, diabetes medications, HTN medications, and inhibitors (angiotensin-converting enzyme inhibitors or angiotensin II receptor antagonists), are represented as binary values (0, 1). The target attribute is a nominal variable labeled “CKD” and “non-CKD”. All attributes in the dataset correspond to the patient’s initial visits in January 2008, except time-year and the binary variable EventCKD35 (represented as 0 and 1). The follow-up duration was extended until June 2017. The binary values 0 and 1 represent patients in non-CKD stages 1 or 2 and CKD stages 3, 4, or 5, respectively. Within the follow-up duration, 56 patients (11.41%) were identified as being in CKD stages 3–5 from the total cohort. In this study’s framework, ‘time’ denotes the length of the follow-up period following the patients’ diagnosis and commencement of therapy, measured in survival months.

### Proposed method

**Figure 2:**
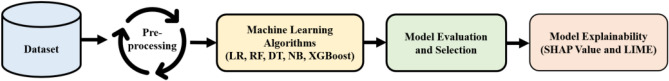
Proposed methodology.

Figure 2 presents a comprehensive framework, illustrating sequential steps from data collection through preprocessing, diverse machine learning algorithms, model evaluation, and the application of explainable AI techniques (LIME and SHAP) for generating local and global explanations.

Initial preprocessing refines raw data, encompassing handling missing values, scaling features, encoding variables, and addressing imbalances. Simultaneously, data partitioning involves creating training and testing subsets. A balanced preprocessing and meticulous data partitioning strategy ensures model robustness, averting overfitting or underfitting. Subsequently, a diverse set of ML algorithms was applied. The overall model underwent evaluation, utilizing both statistical and explainable AI approaches, providing a nuanced assessment of its performance and interpretability.

### Machine learning algorithms

AI, especially ML, has empowered us to organize scattered and unstructured data, becoming a crucial element in business decision-making systems. These ML techniques can extract valuable insights from raw data, laying the foundation for predictive models. Such methods are widely employed in the healthcare sector for predictive analytics and decision support, aiding medical professionals in diagnosing various diseases, among other clinical applications. Numerous studies have leveraged ML for CKD prediction. These studies’ most frequently referenced methods are LR^32^, RF^33^, DT^34^, NB^35^, and XGBoost^36–38^. The following subsections delve into the ML techniques employed for the detection and diagnosis of CKD.

### Explainable artificial intelligence (XAI)

XAI has emerged as a crucial area of research to enhance AI systems’ transparency, accountability, and trustworthiness. XAI aims to mitigate the gap between the “black-box” nature of many ML models and the necessity for understandable explanations of their decision-making processes^39,40^. This is especially relevant in vital fields such as healthcare, finance, and legal systems, where the ability to understand and justify AI-driven decisions is essential. The two most prominent techniques in the XAI field are SHAP and LIME. A comprehensive explanation of these techniques is provided in this subsection.

#### SHapley Additive exPlanations (SHAP)

SHAP is an abbreviation for SHapley Additive exPlanations, first introduced in 2017 by Lundberg and Lee^41^. It employs principles from game theory to provide localized explanations for a model’s predictions. In game theory, the model assumes the role of game rules. At the same time, the input features resemble potential players who can either engage in the game (observed features) or abstain (an invisible feature).

SHAP computes Shapley values by evaluating the model with diverse feature combinations. It measures the average shift in prediction when a feature is active compared to when it is inactive. This estimated variation represents the impact of a feature on the model’s prediction and is known as the Shapley value. So, these Shapley values provide a numerical evaluation of how much each feature aids in the model’s prediction for a given input. SHAP generates Shapley values, which provide model predictions using linear expressions with binary variables to indicate if a particular variable is active in the model^42^.

The SHAP methodology can be outlined as follows: for a given input vector $[x_{1},x_{2},\ldots ,x_{p}]$ comprised of *p* features and a pre-trained model *f*, SHAP can be employed to approximate and yield a more comprehensible model, *g*. This simplified model helps understand how individual features contribute to the overall prediction across all feasible subsets of features. The representation of model *g* is articulated by the subsequent formula^43^:

1 $$\begin{aligned} g(z)=\phi _{0}+\sum _{i=1}^{M}\phi _{i}z_{i} \end{aligned}$$

In Eq. (1), $\phi _{0}$ is the base value of the model, namely the mean value of all model outputs, the sequence $[z_{1},z_{2},\ldots ,z_{p}]$ serves as a streamlined variant of input *x*. In this context, *z* assumes a value of 1 when the related feature plays a role in the prediction and a value of 0 when it is omitted. The coefficient $\phi _{i}\in \mathbb {R}$ signifies the Shapley value attributed to each feature and *M* is the number of features. This value is essentially a weighted average of contributions from all potential feature combinations, where the weights depend on the size of each feature combination. The coefficient $\phi _{i}$ is evaluated by the following expression^43^:

2 $$\begin{aligned} \phi _{i}(f,x)=\sum _{z\subseteq x'}\frac{\left| z \right| !\left( p-\left| z \right| -1 \right) !}{p!}\left[ f_{x}(z)-f_{x}(z \backslash i) \right] \end{aligned}$$

In the given context, $\phi _{i}$ represents the Shapley value associated with feature *i*. The model to be elucidated is denoted by *f*, while *x* signifies the datapoint input. The term $x'$ refers to the distilled data input. The notation $\left| z \right|$ describes the count of non-zero elements in *z*. Additionally, $z\subseteq x'$ encompasses all vectors of *z* where the non-zero components align with the non-zero components present in $x'$. Lastly, *p* indicates the count of streamlined input features. The quantity $f_{x}(z)-f_{x}(z \backslash i)$ represents the deviation of shapley values from their average for each prediction, indicating the contribution of the *i*th variable.

SHAP produces a collection of feature weights that may be used to offer explanations for the model’s predictions. These weights factor in the interplay between features, offering a detailed and refined understanding of the model’s functioning. Essentially, SHAP determines the Shapley value for each feature as a participant within the trained model. This involves evaluating all potential feature combinations, which is time-intensive. However, it’s recognized that SHAP can be efficiently computed for models with tree-like structures.

#### Local interpretable model-agnostic explanations (LIME)

The term LIME is an abbreviation for “local interpretable model-agnostic explanations”. This method, irrespective of the underlying model, is used to investigate the connection between input parameters and the output of a pre-trained model^44^. Fundamentally, LIME operates by approximating the behavior of a complex model near a specific data point. It does so by training a simpler and more interpretable model, often a linear model, on a subset of the original data centered around the instance of interest^45^. The subset of data is formed by changing the instance’s characteristics while keeping the label constant. By observing how the simpler model behaves in these disrupted instances, we can understand how the original model might behave. The explanations offered by LIME for a given observation *x* are represented mathematically as^46^

3 $$\begin{aligned} \phi(x)=\operatorname*{argmin}_{g\in G} L(f,g,\pi_{x})+\Omega(g)\end{aligned}$$

In the given equation, *G* represents the collection of interpretable models, such as linear models and decision trees. The explanation, viewed as a model, is denoted by $g\in G$. Meanwhile, *f* corresponds to the function mapping from $\mathbb {R}^{d}$ to $\mathbb {R}$. The term $\pi _{x}(z)$ signifies the proximity measure between an instance *z* and *x*. The complexity measure of the explanation $g\in G$ is captured by $\Omega (g)$. LIME’s operational approach minimizes the loss function (*L*) without making assumptions about *f*. This is important since LIME’s distinguishing feature is its model-agnostic nature. The size of *L* indicates the difference between the approximation of *f* by *g* in the region described by $\pi _x$, indicating the fidelity of the approximation. The steps involved in the LIME process are generally as follows^43^: (1) Select the specific data points for which an explanation of the model’s prediction is desired. (2) Create new data points by randomly perturbing the features of the selected instance while keeping the label constant. (3) Predict the outcome for each altered data point from the complex black-box model and record the related input features. (4) Train an interpretable model like an LR or DT using the input variables and target values. (5) The coefficients from the interpretable model provide insights into which features exert the most significant impact on the prediction for a given instance.

The key advantage of LIME is its model-agnostic characteristics. It does not matter if you have a decision tree, a neural network, or any other type of classifier; LIME can be applied similarly to explain its predictions. However, it is important to mention that LIME provides local explanations, i.e., individual predictions. It does not necessarily capture the global behavior of the model.

### Performance evaluation

The experimental procedure for implementing ML algorithms was executed exclusively in Python 3.9. The patients were divided into training and testing sets using a random allocation method, with a ratio of 7:3. Furthermore, to improve the performance of ML models, the researchers have incorporated the stratified *k*-fold cross-validation (SKCV) method, as described in Prusty et al.^47^. The primary advantage of SKCV is that it ensures that each fold retains the same proportion of each class as the whole dataset. This reduces the likelihood of creating a fold with very few instances of the minority class. By preserving the class distribution in each fold, SKCV provides a more reliable estimate of the model’s performance. This reduces the variance that might result from non-stratified *k*-fold cross-validation, especially in imbalanced scenarios^48,49^.

In this study, a 10-fold stratified cross-validation (CV) approach was utilized. The training data is first split into 10 folds. The model is subsequently trained on nine folds and validated on the remaining fold. This procedure is repeated ten times with a different fold for validation. The average performance across these iterations indicates the model’s overall generalization capability. The receiver operating characteristic (ROC) curve was used as the assessment measure once the models were finalized to confirm their efficacy further. To ensure the robustness of the ML algorithms, the classification tasks were iterated five times. The performance assessment of the ML model was carried out using a variety of metrics, encompassing accuracy (Acc), sensitivity (Sen), specificity (Spe), and the F-score, as defined in Eqs. (4), (5), (6), and (7), respectively.

4 $$\begin{aligned} \text {Acc}=\frac{t_{p}+t_{n}}{t_{n}+t_{p}+f_{n}+f_{p}} \end{aligned}$$

5 $$\begin{aligned} \text {Sen}=\frac{t_{p}}{f_{n}+t_{p}} \end{aligned}$$

6 $$\begin{aligned} \text {Spe}=\frac{t_{n}}{t_{n}+f_{p}} \end{aligned}$$

7 $$\begin{aligned} \text {F-score}=\frac{2\times t_{p}}{2 \times t_{p}+f_{p}+f_{n}} \end{aligned}$$

Accuracy is a metric that evaluates the classifier’s performance in accurately classifying the different groups. Sensitivity is a quantitative assessment of the accuracy in identifying individuals without CKD, commonly referred to as the true positive rate (TPR) or recall. The true negative rate (TNR), known as specificity, is commonly defined as the proportion of individuals correctly identified as not having CKD. The area under the ROC curve (AUC) is a metric that quantifies the classifier’s capacity to differentiate between distinct classes. The classification process for the ML classifiers was performed using a 10-fold CV approach. This approach was repeated five times to ensure consistency. The reported results include the average classification accuracy from these five repetitions and the highest classification accuracy achieved across all classifiers. Here, the terms $t_{p}$ (true positive), $t_{n}$ (true negative), $f_{p}$ (false positive), and $f_{n}$ (false negative) correspond to the instances that have been accurately predicted as positive, accurately predicted as negative, incorrectly predicted as positive, and incorrectly predicted as negative, respectively.

## Results

The study cohort included 491 patients with $\text {eGFR} \ge 60\,\text {mL}/\text {min}/1.73\,\text {m}^{2}$ (at baseline); 250 males and 241 females in a ratio of 1.04 : 1. The mean age at baseline was $53.20 \pm 13.82$, and their ages range between 23 and 89 years old (median 54, IQR, 44–46). Out of 491 patients, 435 (88.59%) patients are CKD stages 1–2 (non-CKD group), and 56 (11.41%) patients are CKD stages 3–5 (CKD group). The baseline characteristics of the individuals in the nested case-control study are presented in Table 1. eGFR was assessed every 3 months from baseline to June 2017. In this study, CKD stages 3–5 were defined using the National Kidney Foundation Kidney Disease Outcomes Quality Initiative (KDOQI) standards, with an eGFR of less than $60\,\text {mL}/\text {min}/1.73\,\text {m}^{2}$ for $\ge$ 3 months^50^. A descriptive statistical analysis was done using a mean ± SD with an unpaired, two-tailed *t*-test for continuous variables and a frequency distribution for categorical variables (using the chi-squared test) to learn about the patients and their medical conditions. The statistical and quantitative description of the categorical and numerical features is described in Table 1.

**Table 1:** Baseline characteristics of patients.

Variables	Original data (N = 491)	Non-CKD (N = 435)	CKD (N = 56)	*p* value
Age (years)	$53.20\pm 13.82$	$52.04\pm 13.87$	$62.23\pm 9.51$	$< 0.001$
Gender (male (1):female (0))	250:241	214:221	36:20	0.046
History of diabetes (0: false, 1: true)	215 (43.79%)	168 (38.62%)	47 (83.93%)	$< 0.001$
History of coronary heart diseases (0: false, 1: true)	45 (9.16%)	28 (6.44%)	17 (30.36%)	$< 0.001$
History of vascular diseases (0: false, 1: true)	29 (5.91%)	22 (5.06%)	7 (12.50%)	0.036
History of smoking (0: false, 1: true)	75 (15.27%)	61 (14.02%)	14 (25.00%)	0.046
History of hypertension (0: false, 1: true)	335 (68.22%)	284 (65.29%)	51 (91.07%)	$< 0.001$
History of dyslipidemia (0: false, 1: true)	317 (64.56%)	270 (62.07%)	47 (83.93%)	0.001
History of obesity (0: false, 1: true)	248 (50.51%)	217 (49.89%)	31 (55.36%)	0.480
History of ACEIARB use (0: false, 1: true)	219 (44.60%)	176 (40.46%)	43 (76.79%)	$< 0.001$
Dyslipidemia medications (0: false, 1: true)	271 (55.19%)	227 (52.18%)	44 (78.57%)	$< 0.001$
Diabetes medications (0: false, 1: true)	161 (32.79%)	120 (27.59%)	41 (73.21%)	$< 0.001$
Hypertension medication (0: false, 1: true)	303 (61.71%)	256 (58.85%)	47 (83.93%)	$< 0.001$
Cholesterol (mmol/L)	$4.98\pm 1.10$	$5.03\pm 1.09$	$4.54\pm 1.09$	0.002
Triglycerides (mmol/L)	$1.32\pm 0.79$	$1.29\pm 0.80$	$1.50\pm 0.72$	0.054
Glycosylated hemoglobin type A1C (%)	$6.6\pm 1.71$	$6.38\pm 1.44$	$8.30\pm 2.57$	$< 0.001$
Creatinine ($\mu$mol/L)	$67.86\pm 17.92$	$65.93\pm 17.12$	$82.82\pm 17.08$	$< 0.001$
Systolic blood pressure (mmHg)	$131.37\pm 15.69$	$130.69\pm 15.31$	$136.73\pm 17.66$	0.006
Diastolic blood pressure (mmHg)	$76.87\pm 10.71$	$77.14\pm 10.54$	$74.75\pm 11.83$	0.115
Body mass index ($\text {kg}/\text {m}^2$)	$30.18\pm 6.24$	$30.20\pm 6.27$	$30.02\pm 6.06$	0.833

It has been observed from Table 1 that CKD group subjects (stages 3–5) have a higher history of diabetes (83.93% vs 38.62%), CHD (30.36% vs 6.44%), vascular diseases (12.50% vs 5.06%), smoking (25.00% vs 14.02%), HTN (91.07% vs 65.29%), ACEIARB (76.79% vs 40.46%) and the medications of dyslipidemia (78.57% vs 52.18%), diabetes (73.21% vs 27.59%), HTN (83.93% vs 58.85%) than non-CKD group subjects (stages 1–2). The mean age of the non-CKD group ($52.04 \pm 13.87$ years) was significantly lower than that of the CKD group ($62.23 \pm 9.51$ years). The levels of triglycerides (TG), HbA1C, serum creatinine (SCr), and SBP in the CKD group were significantly higher as compared to the non-CKD group. Still, the cholesterol, DBP, and BMI were lower.

**Figure 3:**
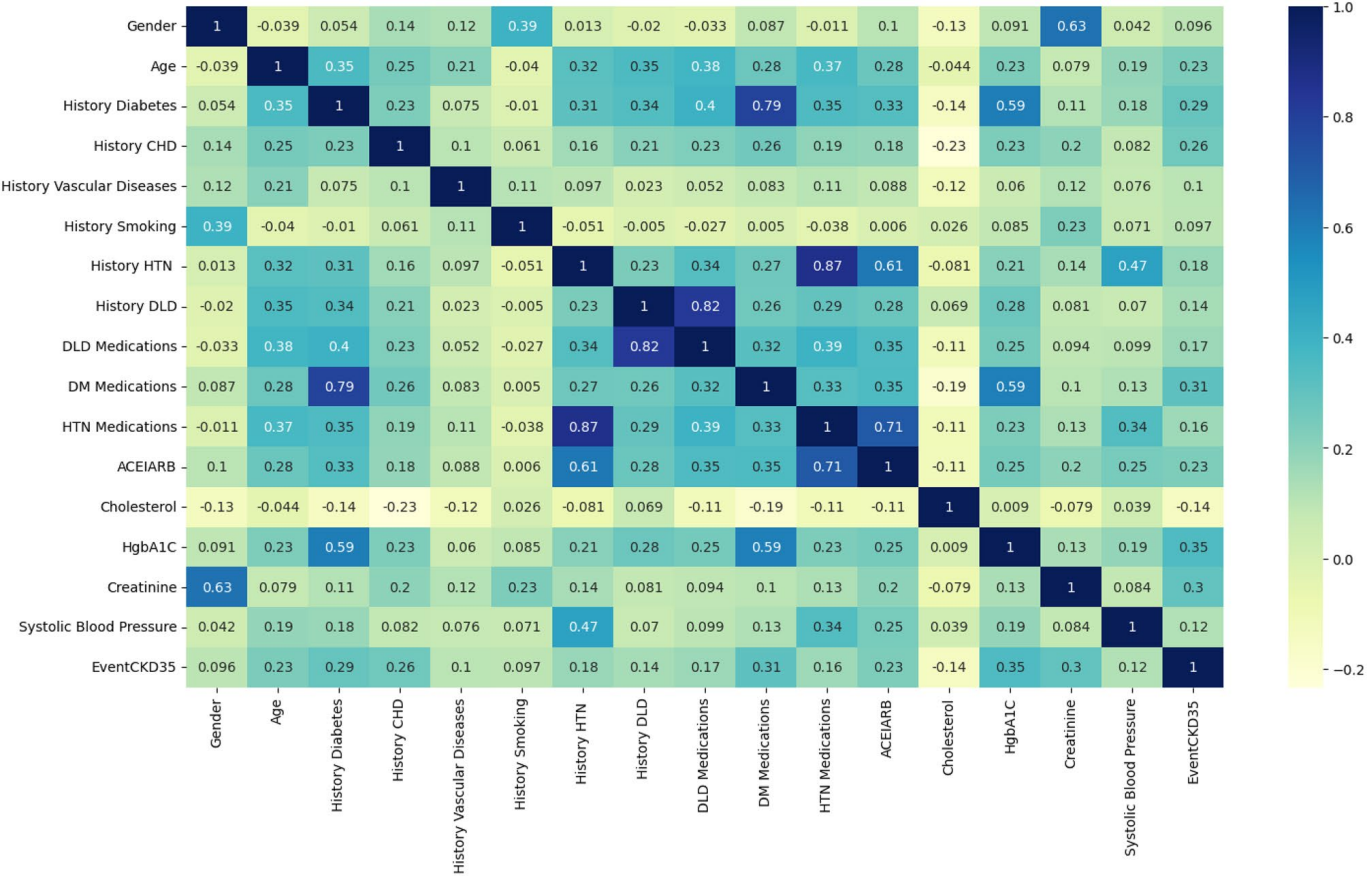
Heatmap of the correlation coefficient matrix. Blue signifies a positive correlation, while yellow represents a negative correlation. The intensity of the color reflects the magnitude of the correlation coefficient, with more vibrant shades indicating stronger correlations. Specifically, shades tending towards blue represent coefficients approaching 1, while those leaning towards yellow represent coefficients approaching − 1.

The data is presented in terms of the mean and SD. The *p* value is a measure used in statistical hypothesis testing to determine the probability of obtaining results as extreme as the ones observed during the study, assuming that the null hypothesis is true. A *p* value of 0.05 or less was regarded as statistically significant. *p* value of the covariates such as age, gender, cholesterol, HgbA1C, creatinine, SBP, history of diabetes, CHD, vascular diseases, smoking, HTN, dyslipidemia, ACEIARB, and medications of dyslipidemia, diabetes, and HTN is less than 0.05, and this indicates that these variables had a significant impact on the CKD stage. The other covariates ($p>$ 0.05) have no significant influence. We identify statistically significant variables and represent their correlations through a heatmap based on the correlation coefficient matrix in Fig. 3.

In Fig. 3, it is evident that gender ($r=0.096$), age ($r=0.230$), history of diabetes ($r=0.290$), history of CHD ($r=0.260$), history of vascular diseases ($r=0.100$), history of smoking ($r=0.097$), history of HTN ($r=0.180$), history of DLD ($r=0.140$), DLD medications ($r=0.170$), DM medications ($r=0.310$), HTN medications ($r=0.160$), ACEIARB ($r=0.230$), HgbA1C ($r=0.350$), creatinine ($r=0.300$), and SBP ($r=0.120$) are positively correlated with the target (EventCKD35), while cholesterol ($r=-0.140$) exhibits a negative correlation with the target. Based on these observations, we inferred that none of the features were redundant and chose to incorporate all of them for model development.

In the study involving 491 qualified participants, it was found that a small proportion of the participants had missing baseline data. Specifically, 3.1% of the participants were missing baseline serum HgbA1C data, equating to approximately 15 individuals. To address these missing values and maintain the integrity of the dataset, we imputed the missing values using the median values of the respective measures. The median, being a robust measure of central tendency, is particularly suitable for handling missing values as it is less sensitive to outliers and variations in the data. Imputing the missing values with the median for HgbA1C ensures that the dataset’s integrity is preserved. This approach allows subsequent analyses and interpretations to be based on a more complete representation of the participants’ profiles. This strategy minimizes the potential bias introduced by missing data and maintains the statistical reliability of the findings, enabling more accurate insights to be drawn from the study. However, the dataset used in this research exhibits inherent imbalances, notably a significant disparity between CKD and non-CKD patients. Specifically, there are relatively few records of CKD patients. To address this imbalance, data augmentation methods such as SMOTE (synthetic minority oversampling technique)^51^ are employed, generating synthetic patient records. This step is crucial to rectify the imbalance and establish a more equitable foundation for ML training. Balancing the dataset becomes imperative for ensuring the robustness and fairness of ML models and their subsequent application in the study. The entire CKD dataset is divided into 10 equal (or nearly equal) parts, or ‘folds’. Each fold should ideally have the same proportion of observations as the whole dataset with respect to the target class. The process consists of 10 iterations. During each iteration, a single fold is designated as the test set. The remaining nine folds are allocated for use as the training set. A model is trained on nine training folds and then tested on a single test fold. The process is iterated until all ten folds have been utilized as the test set. After all ten iterations are completed, the performance metrics from each iteration are averaged to provide an overall assessment of the model’s performance. The hyperparameter configurations for each of the models are outlined in Table 2, with optimization carried out using the grid-search method.

**Table 2:** Hyperparameter evaluation range for the ML models.

ML model	Hyperparameter	Search space	Selected value
LR	C value	0.001, 0.01, 0.1, 1	0.1
	Penalty	‘$\ell _1$’, ‘$\ell _2$’	$\ell _2$
RF	Criterion	‘Gini’, ‘entropy’	‘Entropy’
	Max_depth	10, 20, 30	30
	n_estimators	50, 100, 200	50
	Min_samples_leaf	1, 2, 4	1
	Min_samples_split	2, 5, 10	5
DT	Criterion	‘Gini’, ‘entropy’	‘Gini’
	Max_depth	10, 20, 30, 40	20
	Min_samples_leaf	1, 2, 4	4
	Min_samples_split	2, 5, 10	2
NB	Var_smoothing	Logarithmic scale: 1 to $10^{-9}$, step size = 100	0.0043
XGBoost	n_estimators	50, 100, 200	200
	Max_depth	3, 5, 7	3
	Learning_rate	0.01, 0.05, 0.1	0.05
	Min_child_weight	1, 3, 5	5

**Figure 4:**
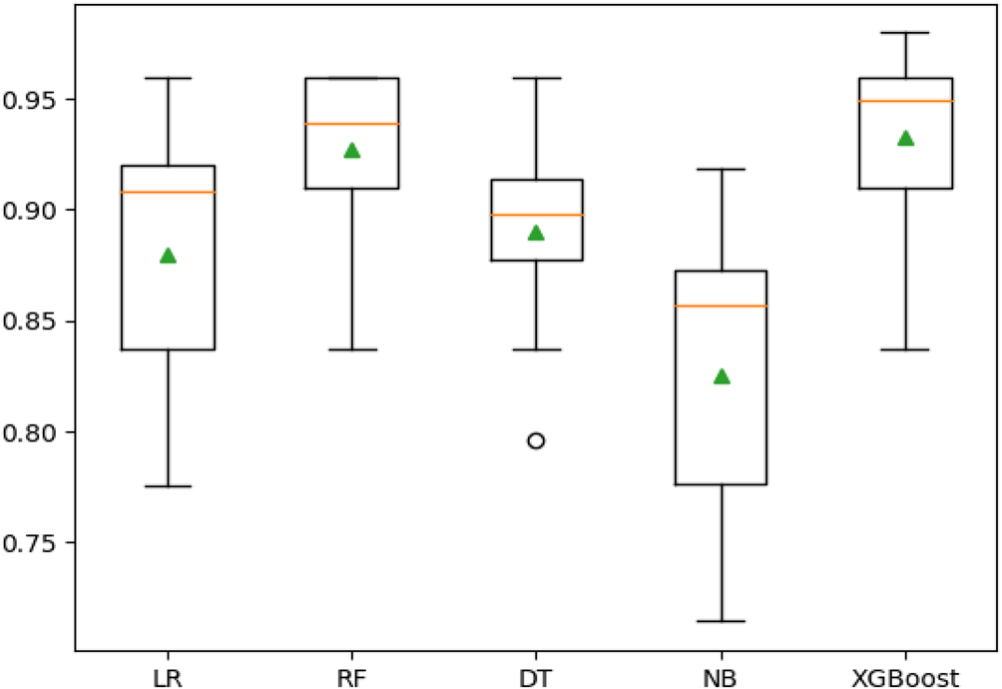
Training performance of the individual algorithms.

The training performance of individual algorithms is visualized in Fig. 4, where LR, RF, DT, and NB achieved accuracy scores of $87.98\%$, $92.67\%$, $89.00\%$, and $82.49\%$, respectively.

In contrast, the state-of-the-art XGBoost algorithm demonstrated superior accuracy at $93.29\%$, with optimal parameters identified through grid-search, including a maximum depth of 3, a learning rate of 0.05, and a minimum child weight of 5. A summary of the performance of the ML models in predicting CKD is outlined in Table 3. For a comprehensive evaluation of these models, key metrics such as sensitivity, specificity, and F-score were calculated, and the results are detailed in the same table. Notably, the XGBoost algorithm exhibited the most impressive overall performance, achieving an AUC score of 0.9689. The AUC scores for other models were LR: 0.9435, RF: 0.9602, DT: 0.9125, and NB: 0.8955 as presented in Table 3.

**Table 3:** Performance of ML models for predicting CKD.

ML Model	Accuracy (%)	Sensitivity (%)	Specificity (%)	F-score	AUC
LR	$87.98 \pm 0.060$	$86.48 \pm 0.089$	$89.37 \pm 0.084$	$0.8768 \pm 0.062$	$0.9435 \pm 0.032$
RF	$92.67 \pm 0.040$	$93.03 \pm 0.041$	$92.68 \pm 0.040$	$0.9286 \pm 0.031$	$0.9602 \pm 0.023$
DT	$89.00 \pm 0.045$	$90.58 \pm 0.069$	$91.29 \pm 0.078$	$0.9113 \pm 0.058$	$0.9125 \pm 0.056$
NB	$82.49 \pm 0.065$	$78.32 \pm 0.095$	$86.68 \pm 0.057$	$0.8144 \pm 0.073$	$0.8955 \pm 0.050$
XGBoost	$93.29 \pm 0.041$	$91.80\pm 0.051$	$94.73 \pm 0.048$	$0.9313 \pm 0.042$	$0.9689 \pm 0.035$

This indicates that the XGBoost algorithm evaluated in this study demonstrated performance comparable to or better than others. This is because the method is designed to build a sequence of tree models one at a time and try to fix the mistakes made by the models before them. This boosting strategy enhances the model’s efficiency. Therefore, it is an efficient ML method built on a scalable end-to-end tree-boosting system design.

**Figure 5:**
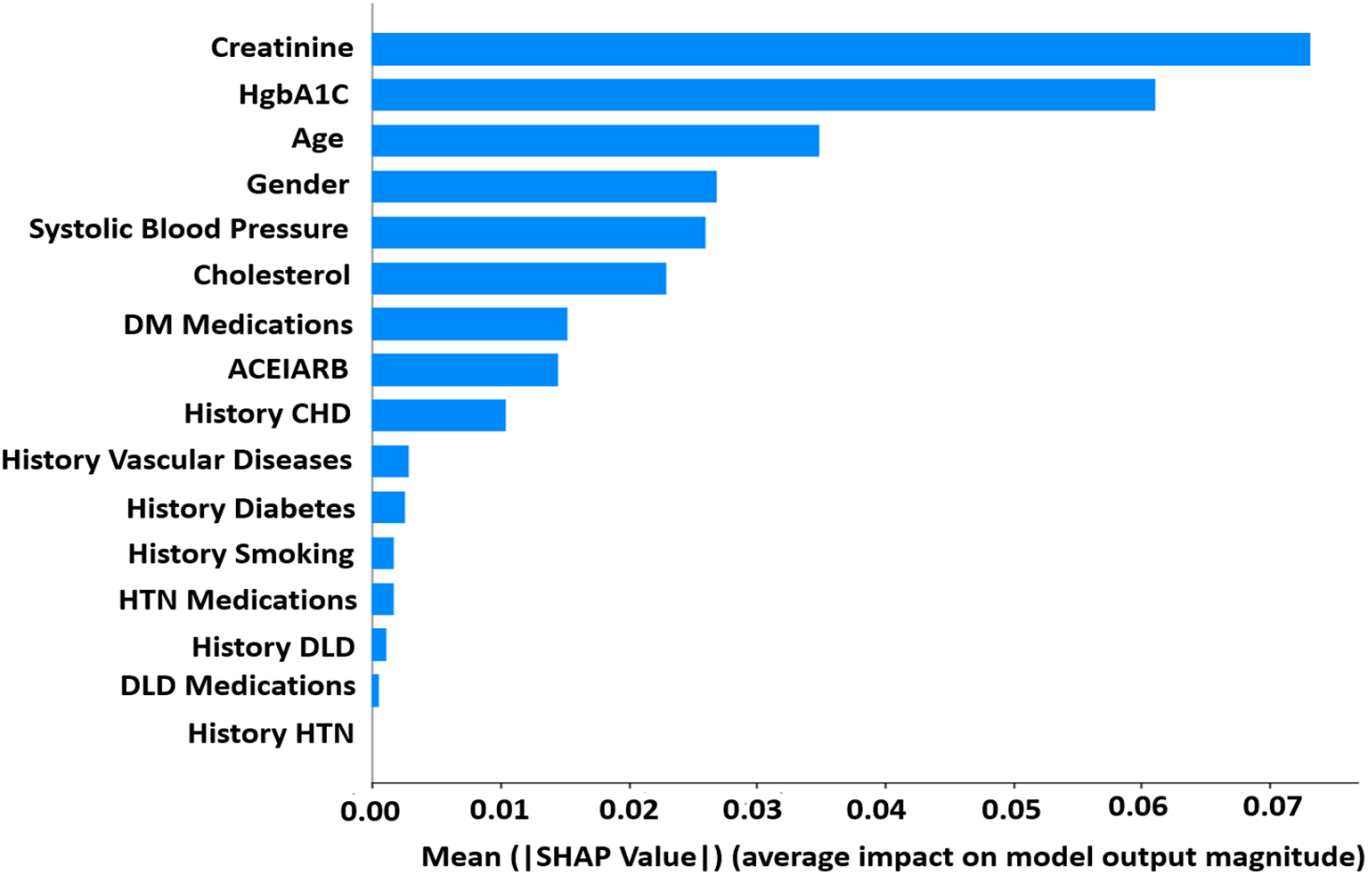
Ranking of feature importance indicated by SHAP algorithm for predicting risk of CKD.

With the explosion of medical data and increased demand for more individualized and precise treatment, the XGBoost algorithm has emerged as a highly promising solution. The impressive execution speed and exceptional model performance of this system position it as a potential frontrunner among ML alternatives. The feature importance ranking with the SHAP summary plot for the XGBoost model is presented in Fig. 5, and the top three most important variables contributing to the predictive model were creatinine, HgbA1C, and age. The figure displays the SHAP value on the *x*-axis, and the feature ranks on the *y*-axis. Features with higher SHAP values are considered more influential in detecting the type and are placed at the top. The features are ordered from highest to lowest SHAP scores in the figure.

**Figure 6:**
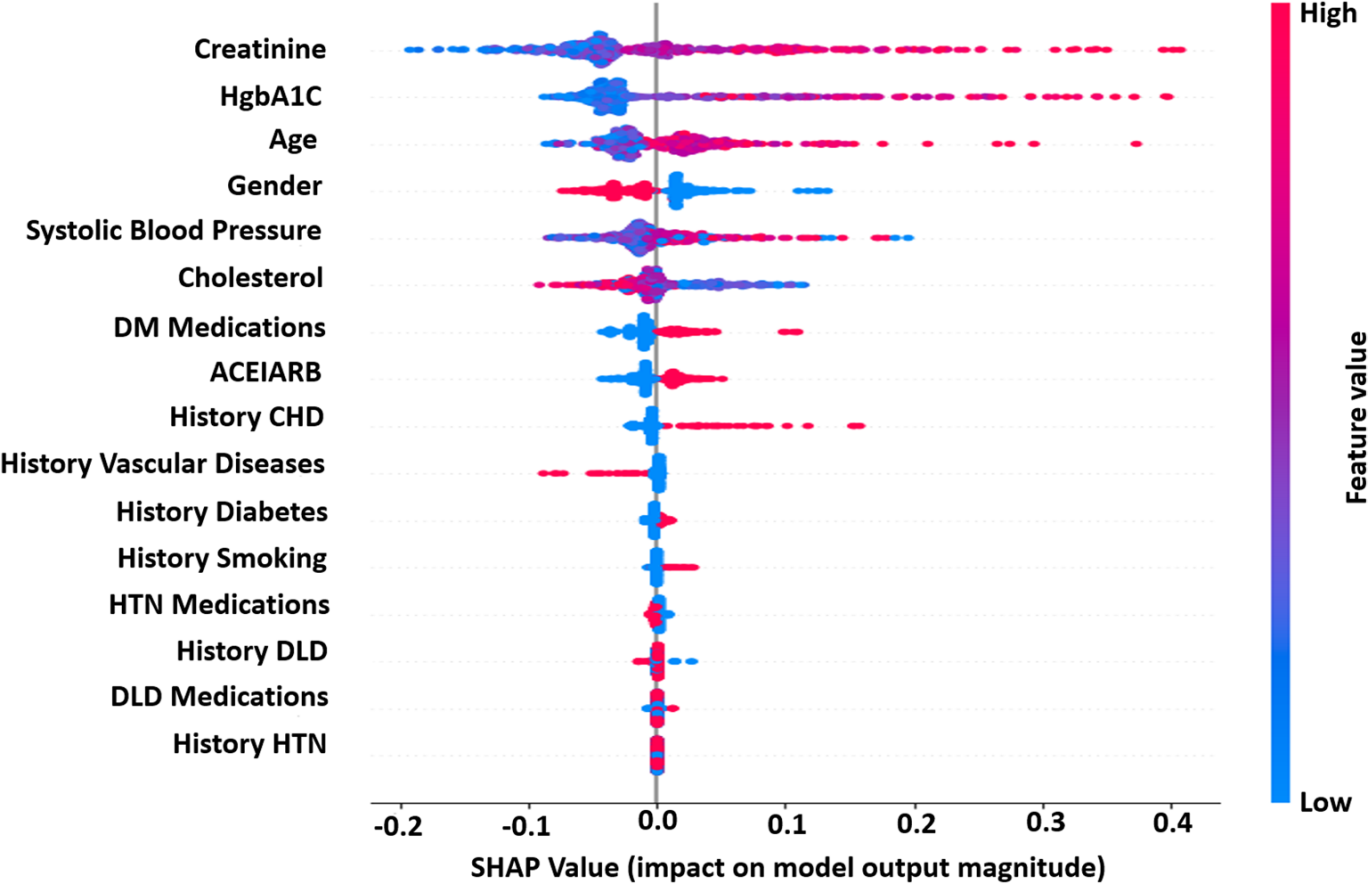
Beeswarm summary plot illustrating the influence of input variables on the predictive performance of the XGBoost model, using SHAP values.

Furthermore, the SHAP beeswarm plot depicted in Fig. 6 offers detailed explanations regarding how the parameters within each variable contribute to the desired outcome (global explanation and interpretation).

**Figure 7:**
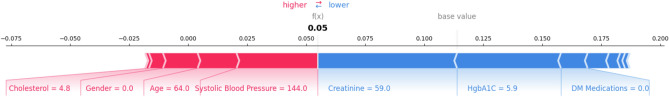
SHAP-based explainable function plot for patient ID 1 (true: non-CKD, predicted: non-CKD).

This visual representation provides insights into the individual contributions of various factors, facilitating a comprehensive understanding of their impact on the model’s predictions. The *x*-axis illustrates the SHAP value, with each line corresponding to a specific feature. Notably, red dots indicate higher feature values, while blue dots denote lower ones. Each point on the plot signifies a single observation, and its position along the *x*-axis reflects the influence of the corresponding feature on the model’s output. We utilized SHAP force analysis and the LIME algorithm to explain the individualized prediction of CKD, drawing two examples from the validation set. The SHAP-based explainable function plot for patient ID 1 is presented in Fig. 7, depicting SHAP values for each feature.

**Figure 8:**
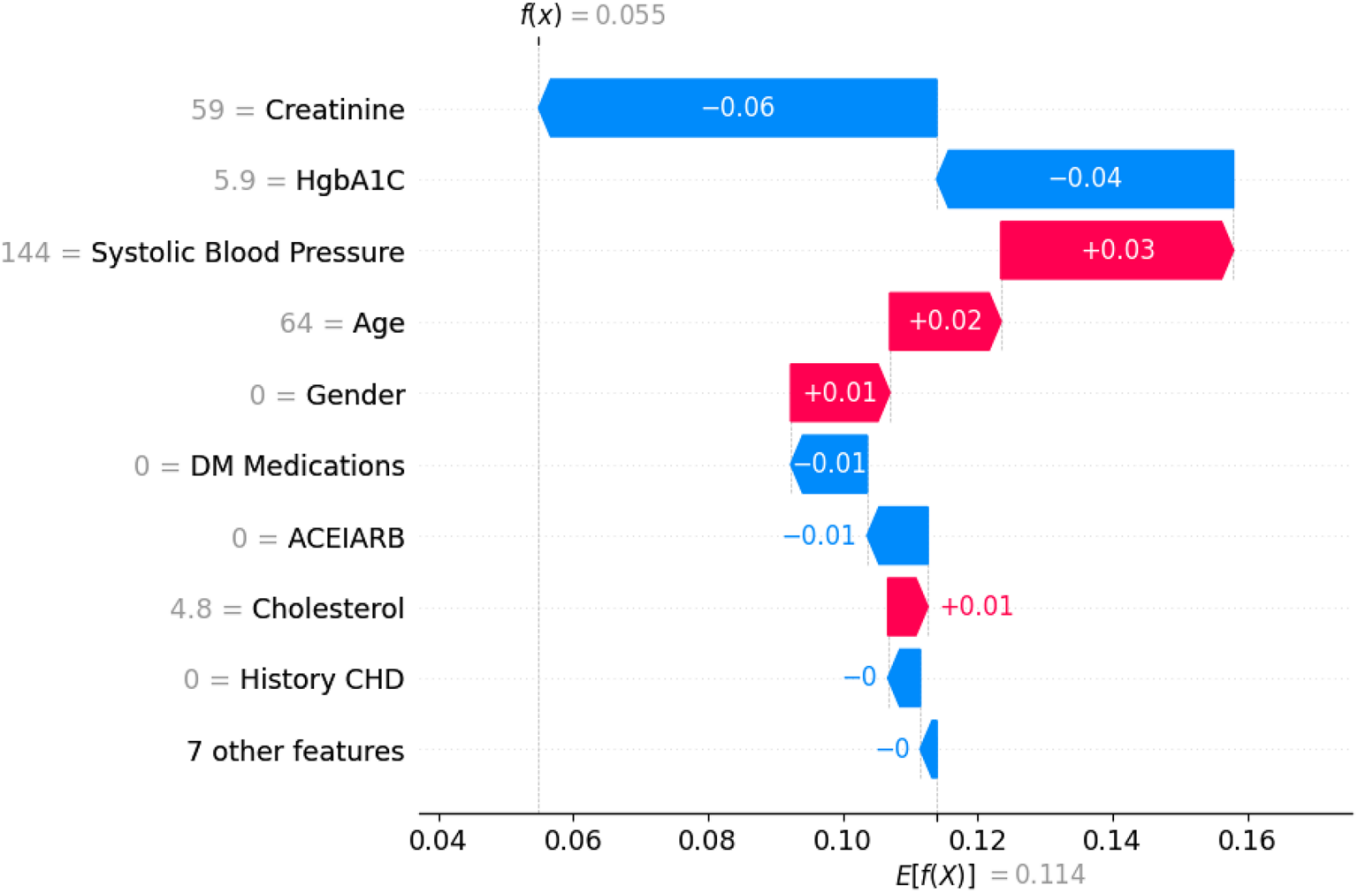
SHAP waterfall plot for patient ID 1 (true: non-CKD, predicted: non-CKD).

The length of each feature’s ‘force’ indicates its impact on the prediction. The impact of a feature on the model’s output is directly proportional to the size of the arrow. According to Fig. 7, the model’s predictive probability values [*f*(*x*)] for CKD patients 1–2 (patient ID: 1) are 0.05. Certain factors, such as systolic blood pressure, the patient’s age (64 years), gender (female), and cholesterol exhibit a positive influence, while creatinine, HgbA1C, and DM medications contribute negatively to predicting the outcome. A visual representation of these contributions can be observed in Fig. 8 through a waterfall plot, depicting the CKD generated by the XGBoost model.

**Figure 9:**
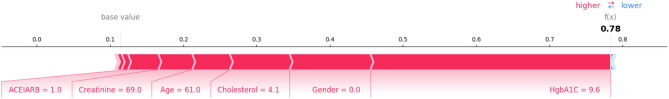
SHAP-based explainable function plot for patient ID 68 (true: CKD, predicted: CKD).

The positive impact of a feature is depicted in red, elevating the prediction from the base value, while the negative effect is shown in blue, lowering the prediction. Each step in the waterfall plot signifies the contribution of a distinct feature to the model’s prediction. The length of each step corresponds to the magnitude of the feature’s impact, and the direction (up or down) indicates whether the feature is driving the prediction higher or lower. As previously mentioned, the label for the target outcome (EventCKD35) indicates a binary result. A value of 0 denotes non-CKD, i.e., CKD stages 1–2, while 1 represents CKD, specifically CKD stages 3–5. Accordingly, the model predicted a probability of 0.05 for patient ID 1, suggesting a low likelihood of CKD stages 3–5. Similarly, in the case of another patient (Patient ID: 68) with $f(x)=0.78$, factors such as HgbA1C, gender (female), cholesterol, age (61 years), creatinine, and ACEIARB play a significant role in predicting a high likelihood of CKD, as illustrated in Fig. 9.

**Figure 10:**
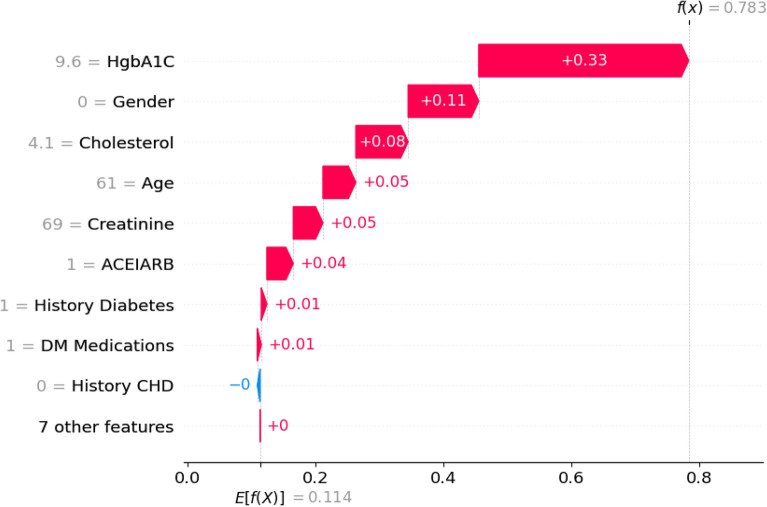
SHAP waterfall plot for patient ID 68 (true: CKD, predicted: CKD).

The waterfall representation in Fig. 10 depicts the prediction of CKD in terms of SHAP values, with features arranged in order of relevance from the most impactful (top) to the least impactful (bottom). Analyzing the SHAP values for Patient ID 68 reveals that the majority of features positively contribute to the final prediction probability of CKD, with HgbA1C being the most influential.

Combining SHAP values from various individual explanations provides a comprehensive perspective on the contribution of features across the entire dataset.

**Figure 11:**
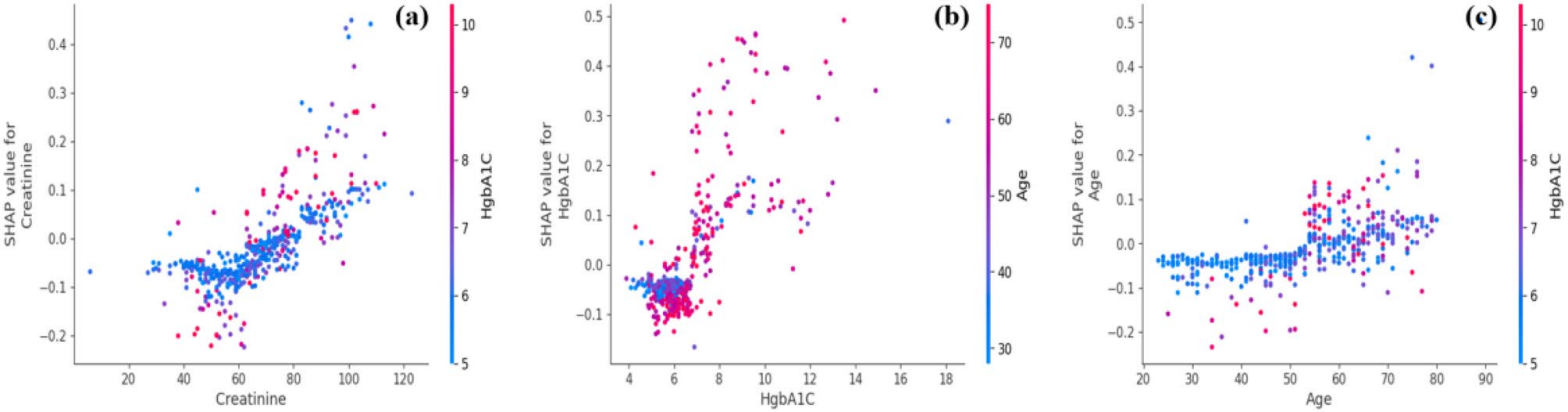
SHAP dependence plot for top three clinical features influencing the XGBoost model (**a**) creatinine; (**b**) HgbA1C; (**c**) age.

Further insight into the top three clinical features contributing to the XGBoost model is presented in the SHAP dependence plot in Fig. 11.

**Figure 12:**
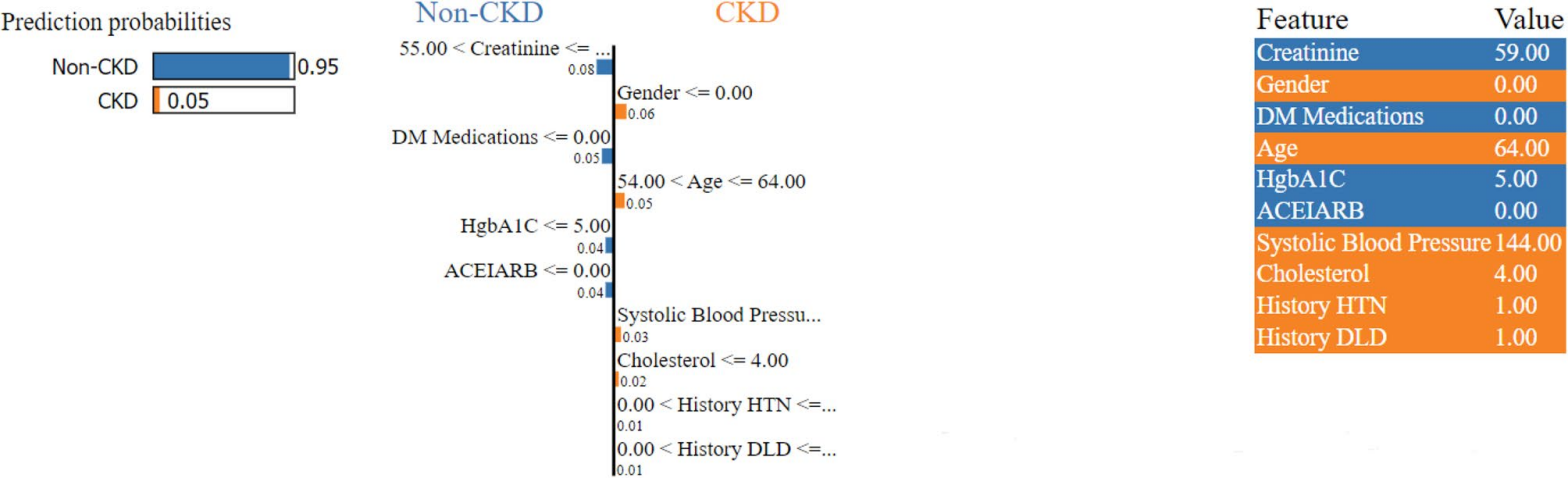
A visualization of LIME model scores for patient ID 1 using the XGBoost model.

**Figure 13:**
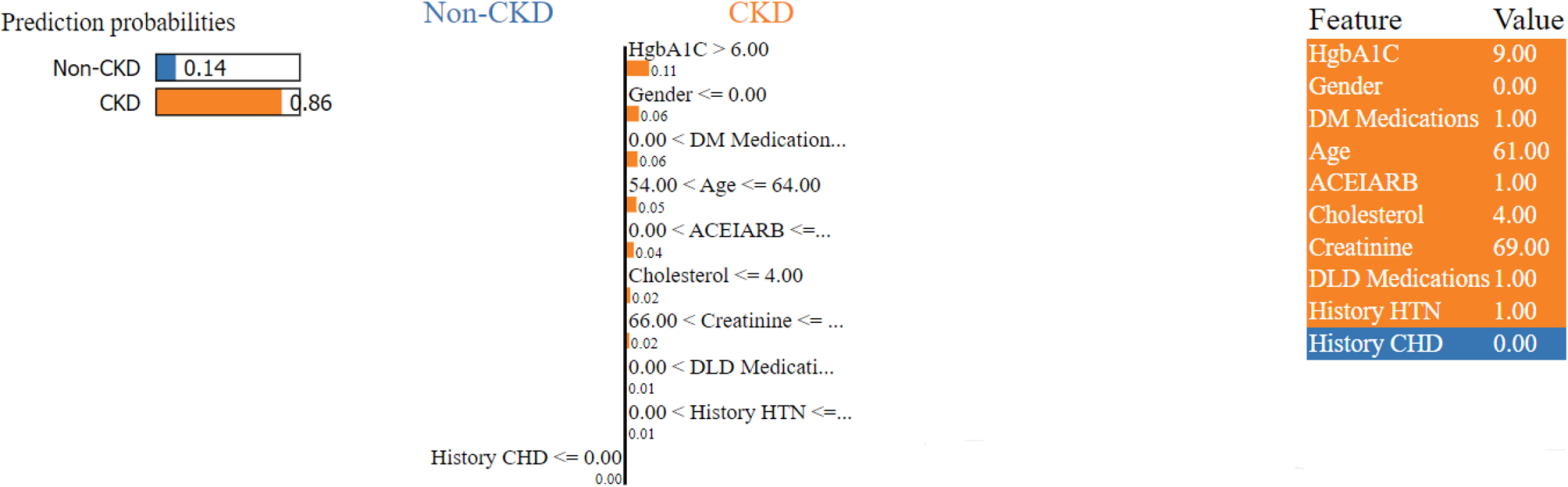
A visualization of LIME model scores for patient ID 68 using the XGBoost model.

This plot visually illustrates the relationship between the values of a single feature and their corresponding SHAP, which represents the impact of a feature value on the model’s prediction. The *x*-axis represents the values of a specific feature, such as creatinine, HgbA1C, and age, while the *y*-axis displays the corresponding SHAP values associated with those feature values. For instance, when examining creatinine, the *x*-axis represents the range of creatinine values, and each point on the plot corresponds to a data point in the dataset. The *y*-axis, representing SHAP values, provides insight into how higher or lower creatinine values influence the model’s prediction. Consistently positive SHAP values for higher creatinine values suggest a positive contribution to the model’s prediction, while consistently negative values imply a negative influence. Similarly, for HgbA1C, the *x*-axis displays the range of HgbA1C values, and the *y*-axis shows the corresponding SHAP values. Positive SHAP values for higher HgbA1C values indicate a positive contribution to the model’s output, while negative values suggest a negative contribution. In the case of age, the *x*-axis represents the range of age values, and the *y*-axis displays the associated SHAP values. Positive SHAP values for higher age values indicate a positive influence on the model’s output, while negative values indicate a negative influence. These SHAP dependence plots help in understanding the relationships between individual clinical features and the model’s predictions. By analyzing these plots, one can determine whether the relationships are linear, nonlinear, or involve complex interactions with other features. Additionally, insights into patterns, concentrations of data points, and information distribution across the plot can be obtained. Overall, examining these plots for creatinine, HgbA1C, and age provides valuable insights into how changes in these clinical features affect the predictions of the XGBoost model, aiding interpretation and potentially informing decision-making in a clinical context. Positive SHAP values signify positive contributions, while negative values indicate negative contributions, offering a nuanced understanding of feature impacts on the model’s output. Furthermore, LIME techniques were employed to analyze and interpret the prediction for a particular instance. This analysis was illustrated by examining an individual patient, as depicted in Figs. 12 and 13. The figure illustrates the features that influenced the categorization of individuals as either CKD (orange) or non-CKD (blue). The specific values of these features are detailed in the figure, representing their respective contributions.

The left section of the figure displays the predicted results for each patient. In the middle part of the figure, the top ten influential variables determining CKD and non-CKD events are highlighted in descending order of importance; the length of each bar signifies the weight or significance of that variable in the prediction process. Attributes of the orange color support CKD, and attributes of the blue color support non-CKD. The floating-point numbers displayed on the horizontal bars indicate the relative significance of these features. A longer bar indicates a variable with a more pronounced influence on the outcome. The rightmost section of the figure shows the critical values of these ten variables at their peak influence on the outcome. The prediction outcome for patient ID 1, as illustrated in Fig. 12, confidently suggests that this specific patient is at CKD stages 1–2, with a prediction confidence of 95%. Similarly, Fig. 13 shows the prediction for patient ID 68, categorizing this individual as non-CKD, specifically falling within CKD stages 3–5, with a prediction confidence of 86%. Moreover, the figures elaborate on the reasoning behind these predictions by highlighting the contributions of the input features to the projected outcomes.

Using the SHAP and LIME approaches, we assessed the prediction probabilities of the models for each patient in our database, which included 491 cases. These assessments provide in-depth knowledge regarding how the models recognize each patient and the chance that they will exhibit particular outcomes or attributes. The supplemental Table S1 presents a more in-depth analysis and context for the results. The predictive probabilities correlate with patients with an elevated likelihood of experiencing complications stemming from their kidney ailment, yielding higher scores for those at greater risk. Conversely, lower risk scores indicate a reduced likelihood of encountering such complications. This outcome in predictive probabilities proves beneficial for healthcare practitioners, as it aids in discerning patients categorized under CKD stages 1–2 or 3–5. By understanding these probabilities, professionals can pinpoint patients needing more intensive monitoring, tailored interventions, or specialized care, depending on their specific risk profiles.

## Discussion

This study presents the development and testing of five ML models. We trained and evaluated these models using a dataset that included 22 clinical features. This research aimed to predict patients that fall into two categories: CKD stages 1–2 or 3–5. The XGBoost model outperformed other models such as LR, RF, DT, and NB. Furthermore, in response to the continuous criticism of ML models for their frequently opaque and difficult-to-understand predictions, we have added explainability and interpretability characteristics to our XGBoost model, utilizing the LIME and SHAP techniques. These techniques provide a detailed analysis of patient-specific information regarding the impact of each variable on the predicted chance of CKD as determined by the model. This analysis includes a local interpretation, which focuses on how each variable contributes to the chance of CKD for a specific patient.

In the past, various ML algorithms have been utilized to effectively and accurately diagnose patients with CKD. However, a current trend is emerging to explore the applicability of ML for diagnosis and prognosis in CKD. In a recent study, Liu et al. discovered that the XGBoost model outperformed other machine learning models such as LR, RF, and SVM in predicting mortality among patients with acute renal injury^52^. Similarly, Hu et al. found that XGBoost outperformed SVM, KNN, LR, DT, NB, and RF^53^. Moreover, a recent meta-analysis conducted by Song et al. has confirmed that XGBoost outperformed other machine learning techniques, such as SVM and Bayesian networks, in predicting acute kidney injury outcomes^54^. However, these studies lacked external validation and explainability. Addressing this research gap, our research focused on the potential of ML algorithms for prognostication in CKD. Furthermore, the LIME and SHAP techniques were utilized to further improve the transparency and interpretability of our model’s predictions. In addition to determining the predictive performance of the XGBoost model, the LIME and SHAP techniques provide valuable insights into the rationale behind the model’s predictions. In particular, SHAP values were used to demonstrate the importance of features and how certain compound sub-structures affect XGBoost’s predictions.

Among the variables evaluated, creatinine, HgbA1C, and age emerged as the three most important contributors to the XGBoost model. By incorporating three key features—creatinine, HgbA1C, and age—the model developed in this study demonstrates significant clinical applicability. These features have the potential to greatly assist physicians in diagnosing patients with CKD promptly and accurately. Creatinine, functioning as a reliable marker for kidney health, contributes to the model’s ability to enhance diagnostic precision. Another crucial feature, HgbA1C, plays a pivotal role in assessing long-term blood glucose control. Elevated HgbA1C levels signal poorly managed diabetes, a significant risk factor for CKD. Additionally, including age in the model recognizes the natural decline in kidney function associated with aging, offering a demographic perspective on CKD risk. By incorporating these significant features, the model strives to provide a more accurate and timely assessment of patients’ kidney health, potentially leading to improved management and outcomes for individuals at risk or already affected by CKD.

## Conclusion

This study has introduced a robust ML framework leveraging computational intelligence for predictive diagnosis and variable significance determination in CKD. Various ML models, including LR, RF, NB, DT, and XGBoost, were employed. Among them, the XGBoost model emerged as the top performer, achieving an impressive AUC of 0.9689 and an accuracy of $93.29\%$ in CKD prediction. Additionally, the investigation employed interpretable ML techniques such as SHAP and LIME for further analysis, providing insights into model behavior on both local and global scales. These methodologies contributed coherent and rational explanations, thereby enhancing the overall transparency of the model. To further improve this system, future enhancements could involve integrating more comprehensive healthcare datasets and incorporating advanced deep learning (DL) algorithms.

### Supplementary Information

Supplementary Information.

## Data Availability

The datasets utilized for this study can be accessed freely at the link below: https://figshare.com/articles/dataset/6711155?file=12242270.
