# Supplementary material for: Investigation on explainable machine learning models to predict chronic kidney diseases
Source: Sci Rep. 2024 Feb 14;14:3687. doi: 10.1038/s41598-024-54375-4 (PMC10866953; doi:10.1038/s41598-024-54375-4)
Supplement: Supplementary file 1 — Supplementary Information. [file 41598_2024_54375_MOESM1_ESM.docx]

**Supplementary Material**

**Supplemental Table S1:** Predictive Probabilities of Complications in Patients with Chronic Kidney Diseases

| **Patient ID** | **LIME (Non-CKD)** | **LIME (CKD)** | **SHAP** |
| --- | --- | --- | --- |
|  | **Predictive Probability** | **Predicted Probability** | $\left\vert\boldsymbol{f(x)} \right\vert$ |
| 1 | 0.95 | 0.05 | 0.05 |
| 2 | 0.99 | 0.01 | 0.03 |
| 3 | 0.99 | 0.01 | 0.01 |
| 4 | 0.97 | 0.03 | 0.02 |
| 5 | 0.87 | 0.13 | 0.14 |
| 6 | 0.95 | 0.05 | 0.00 |
| 7 | 0.98 | 0.02 | 0.03 |
| 8 | 0.98 | 0.02 | 0.01 |
| 9 | 0.98 | 0.02 | 0.02 |
| 10 | 0.98 | 0.02 | 0.08 |
| 11 | 0.98 | 0.02 | 0.02 |
| 12 | 0.84 | 0.16 | 0.10 |
| 13 | 0.98 | 0.02 | 0.01 |
| 14 | 0.91 | 0.09 | 0.28 |
| 15 | 0.97 | 0.03 | 0.04 |
| 16 | 0.97 | 0.03 | 0.02 |
| 17 | 0.99 | 0.01 | 0.01 |
| 18 | 0.99 | 0.01 | 0.08 |
| 19 | 0.97 | 0.03 | 0.14 |
| 20 | 0.96 | 0.04 | 0.07 |
| 21 | 0.90 | 0.10 | 0.33 |
| 22 | 0.46 | 0.54 | 0.58 |
| 23 | 0.99 | 0.01 | 0.01 |
| 24 | 0.99 | 0.01 | 0.01 |
| 25 | 1.00 | 0.00 | 0.02 |
| 26 | 0.91 | 0.09 | 0.07 |
| 27 | 0.98 | 0.02 | 0.09 |
| 28 | 0.99 | 0.01 | 0.00 |
| 29 | 0.75 | 0.25 | 0.71 |
| 30 | 0.94 | 0.06 | 0.13 |
| 31 | 0.92 | 0.08 | 0.07 |
| 32 | 0.91 | 0.09 | 0.18 |
| 33 | 0.98 | 0.02 | 0.02 |
| 34 | 0.95 | 0.05 | 0.21 |
| 35 | 0.94 | 0.06 | 0.03 |
| 36 | 0.99 | 0.01 | 0.02 |
| 37 | 0.99 | 0.01 | 0.02 |
| 38 | 0.97 | 0.03 | 0.05 |
| 39 | 0.88 | 0.12 | 0.10 |
| 40 | 0.90 | 0.10 | 0.05 |
| 41 | 0.97 | 0.03 | 0.01 |
| 42 | 0.99 | 0.01 | 0.02 |
| 43 | 0.97 | 0.03 | 0.03 |
| 44 | 0.99 | 0.01 | 0.01 |
| 45 | 0.98 | 0.02 | 0.01 |
| 46 | 0.94 | 0.06 | 0.01 |
| 47 | 0.98 | 0.02 | 0.02 |
| 48 | 0.99 | 0.01 | 0.04 |
| 49 | 0.97 | 0.03 | 0.10 |
| 50 | 0.97 | 0.03 | 0.05 |
| 51 | 0.99 | 0.01 | 0.02 |
| 52 | 0.98 | 0.02 | 0.03 |
| 53 | 0.94 | 0.06 | 0.02 |
| 54 | 0.78 | 0.22 | 0.12 |
| 55 | 0.98 | 0.02 | 0.02 |
| 56 | 0.74 | 0.26 | 0.66 |
| 57 | 0.98 | 0.02 | 0.09 |
| 58 | 0.99 | 0.01 | 0.03 |
| 59 | 0.92 | 0.08 | 0.14 |
| 60 | 0.99 | 0.01 | 0.01 |
| 61 | 0.98 | 0.02 | 0.01 |
| 62 | 0.99 | 0.01 | 0.02 |
| 63 | 0.96 | 0.04 | 0.07 |
| 64 | 0.99 | 0.01 | 0.00 |
| 65 | 0.98 | 0.02 | 0.02 |
| 66 | 0.98 | 0.02 | 0.02 |
| 67 | 0.98 | 0.02 | 0.01 |
| 68 | 0.14 | 0.86 | 0.78 |
| 69 | 0.95 | 0.05 | 0.10 |
| 70 | 0.38 | 0.62 | 1.08 |
| 71 | 0.99 | 0.01 | 0.04 |
| 72 | 0.99 | 0.01 | 0.02 |
| 73 | 0.99 | 0.01 | 0.05 |
| 74 | 0.95 | 0.05 | 0.06 |
| 75 | 0.99 | 0.01 | 0.02 |
| 76 | 0.85 | 0.15 | 0.03 |
| 77 | 0.99 | 0.01 | 0.01 |
| 78 | 0.94 | 0.06 | 0.10 |
| 79 | 1.00 | 0.00 | 0.02 |
| 80 | 0.94 | 0.06 | 0.02 |
| 81 | 0.84 | 0.16 | 0.37 |
| 82 | 0.99 | 0.01 | 0.01 |
| 83 | 0.75 | 0.25 | 0.19 |
| 84 | 0.13 | 0.87 | 0.84 |
| 85 | 0.97 | 0.03 | 0.04 |
| 86 | 0.73 | 0.27 | 0.14 |
| 87 | 0.75 | 0.25 | 0.04 |
| 88 | 0.40 | 0.60 | 0.60 |
| 89 | 0.43 | 0.57 | 0.33 |
| 90 | 0.88 | 0.12 | 0.08 |
| 91 | 0.91 | 0.09 | 0.29 |
| 92 | 0.96 | 0.04 | 0.01 |
| 93 | 0.99 | 0.01 | 0.02 |
| 94 | 0.99 | 0.01 | 0.01 |
| 95 | 0.89 | 0.11 | 0.73 |
| 96 | 0.98 | 0.02 | 0.03 |
| 97 | 0.21 | 0.79 | 0.79 |
| 98 | 0.99 | 0.01 | 0.02 |
| 99 | 1.00 | 0.00 | 0.01 |
| 100 | 0.95 | 0.05 | 0.00 |
| 101 | 0.98 | 0.02 | 0.01 |
| 102 | 0.94 | 0.06 | 0.02 |
| 103 | 0.97 | 0.03 | 0.04 |
| 104 | 0.98 | 0.02 | 0.03 |
| 105 | 0.99 | 0.01 | 0.01 |
| 106 | 0.90 | 0.10 | 0.02 |
| 107 | 0.98 | 0.02 | 0.01 |
| 108 | 0.80 | 0.20 | 0.52 |
| 109 | 0.84 | 0.16 | 0.23 |
| 110 | 0.98 | 0.02 | 0.02 |
| 111 | 0.95 | 0.05 | 0.03 |
| 112 | 0.72 | 0.28 | 0.57 |
| 113 | 1.00 | 0.00 | 0.01 |
| 114 | 0.93 | 0.07 | 0.05 |
| 115 | 0.97 | 0.03 | 0.04 |
| 116 | 0.68 | 0.32 | 0.46 |
| 117 | 0.99 | 0.01 | 0.01 |
| 118 | 0.78 | 0.22 | 0.13 |
| 119 | 0.99 | 0.01 | 0.02 |
| 120 | 0.96 | 0.04 | 0.07 |
| 121 | 0.99 | 0.01 | 0.01 |
| 122 | 0.89 | 0.11 | 0.06 |
| 123 | 0.98 | 0.02 | 0.01 |
| 124 | 0.77 | 0.23 | 0.16 |
| 125 | 0.99 | 0.01 | 0.01 |
| 126 | 0.97 | 0.03 | 0.02 |
| 127 | 0.73 | 0.27 | 0.11 |
| 128 | 0.92 | 0.08 | 0.03 |
| 129 | 0.96 | 0.04 | 0.03 |
| 130 | 0.95 | 0.05 | 0.01 |
| 131 | 1.00 | 0.00 | 0.02 |
| 132 | 0.99 | 0.01 | 0.08 |
| 133 | 0.65 | 0.35 | 0.11 |
| 134 | 0.95 | 0.05 | 0.11 |
| 135 | 0.99 | 0.01 | 0.02 |
| 136 | 0.99 | 0.01 | 0.03 |
| 137 | 0.99 | 0.01 | 0.01 |
| 138 | 0.97 | 0.03 | 0.05 |
| 139 | 1.00 | 0.00 | 0.01 |
| 140 | 0.95 | 0.05 | 0.08 |
| 141 | 0.96 | 0.04 | 0.04 |
| 142 | 0.57 | 0.43 | 0.20 |
| 143 | 0.97 | 0.03 | 0.02 |
| 144 | 0.98 | 0.02 | 0.01 |
| 145 | 0.98 | 0.02 | 0.01 |
| 146 | 0.96 | 0.04 | 0.14 |
| 147 | 0.80 | 0.20 | 0.20 |
| 148 | 1.00 | 0.00 | 0.01 |
| 149 | 0.68 | 0.32 | 0.23 |
| 150 | 0.97 | 0.03 | 0.05 |
| 151 | 0.98 | 0.02 | 0.01 |
| 152 | 0.99 | 0.01 | 0.01 |
| 153 | 0.97 | 0.03 | 0.04 |
| 154 | 0.99 | 0.01 | 0.00 |
| 155 | 0.95 | 0.05 | 0.04 |
| 156 | 1.00 | 0.00 | 0.00 |
| 157 | 0.66 | 0.34 | 0.13 |
| 158 | 0.20 | 0.80 | 0.62 |
| 159 | 0.99 | 0.01 | 0.01 |
| 160 | 0.97 | 0.03 | 0.02 |
| 161 | 0.99 | 0.01 | 0.01 |
| 162 | 0.97 | 0.04 | 0.08 |
| 163 | 0.99 | 0.01 | 0.01 |
| 164 | 0.64 | 0.36 | 0.34 |
| 165 | 0.99 | 0.01 | 0.04 |
| 166 | 0.94 | 0.06 | 0.03 |
| 167 | 0.99 | 0.01 | 0.00 |
| 168 | 0.97 | 0.03 | 0.12 |
| 169 | 0.99 | 0.01 | 0.01 |
| 170 | 0.92 | 0.08 | 0.06 |
| 171 | 0.93 | 0.07 | 0.18 |
| 172 | 0.63 | 0.37 | 0.07 |
| 173 | 0.97 | 0.03 | 0.04 |
| 174 | 0.70 | 0.30 | 0.68 |
| 175 | 0.98 | 0.02 | 0.01 |
| 176 | 0.97 | 0.03 | 0.02 |
| 177 | 0.98 | 0.02 | 0.01 |
| 178 | 1.00 | 0.00 | 0.02 |
| 179 | 0.98 | 0.02 | 0.04 |
| 180 | 0.73 | 0.27 | 0.14 |
| 181 | 0.98 | 0.04 | 0.15 |
| 182 | 0.72 | 0.28 | 0.16 |
| 183 | 0.99 | 0.01 | 0.01 |
| 184 | 0.98 | 0.02 | 0.02 |
| 185 | 1.00 | 0.00 | 0.01 |
| 186 | 0.98 | 0.02 | 0.01 |
| 187 | 0.99 | 0.01 | 0.01 |
| 188 | 0.96 | 0.04 | 0.07 |
| 189 | 0.99 | 0.01 | 0.01 |
| 190 | 0.98 | 0.02 | 0.06 |
| 191 | 0.99 | 0.01 | 0.01 |
| 192 | 0.97 | 0.03 | 0.02 |
| 193 | 0.99 | 0.01 | 0.02 |
| 194 | 0.96 | 0.04 | 0.15 |
| 195 | 0.98 | 0.02 | 0.01 |
| 196 | 0.97 | 0.03 | 0.00 |
| 197 | 0.89 | 0.11 | 0.38 |
| 198 | 0.98 | 0.02 | 0.02 |
| 199 | 0.98 | 0.02 | 0.01 |
| 200 | 0.99 | 0.01 | 0.01 |
| 201 | 0.98 | 0.02 | 0.02 |
| 202 | 0.96 | 0.04 | 0.04 |
| 203 | 0.99 | 0.01 | 0.00 |
| 204 | 0.99 | 0.01 | 0.02 |
| 205 | 0.96 | 0.04 | 0.09 |
| 206 | 0.93 | 0.07 | 0.11 |
| 207 | 0.53 | 0.47 | 0.52 |
| 208 | 0.96 | 0.04 | 0.07 |
| 209 | 0.88 | 0.12 | 0.18 |
| 210 | 1.00 | 0.00 | 0.02 |
| 211 | 1.00 | 0.00 | 0.02 |
| 212 | 0.94 | 0.06 | 0.03 |
| 213 | 0.96 | 0.04 | 0.02 |
| 214 | 0.98 | 0.02 | 0.01 |
| 215 | 0.48 | 0.52 | 0.48 |
| 216 | 0.99 | 0.01 | 0.01 |
| 217 | 0.90 | 0.10 | 0.01 |
| 218 | 1.00 | 0.00 | 0.01 |
| 219 | 0.98 | 0.02 | 0.03 |
| 220 | 1.00 | 0.00 | 0.01 |
| 221 | 0.99 | 0.01 | 0.01 |
| 222 | 0.98 | 0.02 | 0.03 |
| 223 | 0.63 | 0.37 | 0.02 |
| 224 | 0.90 | 0.10 | 0.09 |
| 225 | 0.93 | 0.07 | 0.43 |
| 226 | 0.98 | 0.02 | 0.01 |
| 227 | 0.64 | 0.36 | 0.21 |
| 228 | 0.92 | 0.08 | 0.02 |
| 229 | 0.33 | 0.67 | 0.59 |
| 230 | 0.92 | 0.08 | 0.13 |
| 231 | 0.99 | 0.01 | 0.01 |
| 232 | 1.00 | 0.00 | 0.00 |
| 233 | 0.97 | 0.03 | 0.00 |
| 234 | 0.99 | 0.01 | 0.02 |
| 235 | 0.99 | 0.01 | 0.01 |
| 236 | 0.97 | 0.03 | 0.03 |
| 237 | 0.98 | 0.02 | 0.06 |
| 238 | 0.97 | 0.03 | 0.12 |
| 239 | 0.86 | 0.14 | 0.04 |
| 240 | 1.00 | 0.00 | 0.01 |
| 241 | 0.96 | 0.04 | 0.05 |
| 242 | 0.53 | 0.47 | 0.62 |
| 243 | 0.98 | 0.02 | 0.02 |
| 244 | 0.99 | 0.01 | 0.01 |
| 245 | 0.91 | 0.09 | 0.05 |
| 246 | 0.58 | 0.42 | 0.86 |
| 247 | 0.97 | 0.03 | 0.03 |
| 248 | 0.96 | 0.04 | 0.04 |
| 249 | 0.99 | 0.01 | 0.00 |
| 250 | 0.24 | 0.76 | 0.79 |
| 251 | 0.61 | 0.39 | 0.22 |
| 252 | 0.98 | 0.02 | 0.01 |
| 253 | 0.97 | 0.03 | 0.08 |
| 254 | 0.85 | 0.15 | 0.11 |
| 255 | 1.00 | 0.00 | 0.01 |
| 256 | 0.98 | 0.02 | 0.00 |
| 257 | 0.97 | 0.03 | 0.02 |
| 258 | 0.97 | 0.03 | 0.02 |
| 259 | 0.60 | 0.40 | 0.28 |
| 260 | 0.99 | 0.01 | 0.02 |
| 261 | 0.96 | 0.04 | 0.01 |
| 262 | 0.76 | 0.24 | 0.09 |
| 263 | 0.97 | 0.03 | 0.01 |
| 264 | 0.74 | 0.26 | 0.15 |
| 265 | 0.80 | 0.20 | 0.20 |
| 266 | 0.87 | 0.13 | 0.17 |
| 267 | 0.98 | 0.02 | 0.06 |
| 268 | 0.97 | 0.03 | 0.02 |
| 269 | 0.84 | 0.16 | 0.05 |
| 270 | 0.57 | 0.43 | 0.22 |
| 271 | 0.91 | 0.09 | 0.14 |
| 272 | 0.99 | 0.01 | 0.02 |
| 273 | 0.98 | 0.02 | 0.05 |
| 274 | 0.91 | 0.09 | 0.07 |
| 275 | 0.99 | 0.01 | 0.00 |
| 276 | 0.99 | 0.01 | 0.01 |
| 277 | 0.98 | 0.02 | 0.07 |
| 278 | 0.85 | 0.15 | 0.08 |
| 279 | 0.99 | 0.01 | 0.01 |
| 280 | 0.93 | 0.07 | 0.18 |
| 281 | 0.99 | 0.01 | 0.01 |
| 282 | 0.31 | 0.69 | 0.89 |
| 283 | 0.83 | 0.17 | 0.15 |
| 284 | 0.93 | 0.07 | 0.04 |
| 285 | 0.83 | 0.17 | 0.12 |
| 286 | 0.96 | 0.04 | 0.06 |
| 287 | 0.99 | 0.01 | 0.01 |
| 288 | 0.96 | 0.04 | 0.12 |
| 289 | 0.87 | 0.13 | 0.08 |
| 290 | 0.99 | 0.01 | 0.02 |
| 291 | 0.78 | 0.22 | 0.18 |
| 292 | 0.97 | 0.03 | 0.03 |
| 293 | 0.95 | 0.05 | 0.01 |
| 294 | 0.99 | 0.01 | 0.01 |
| 295 | 0.76 | 0.24 | 0.12 |
| 296 | 0.25 | 0.75 | 0.54 |
| 297 | 0.98 | 0.02 | 0.06 |
| 298 | 0.99 | 0.01 | 0.03 |
| 299 | 0.94 | 0.06 | 0.03 |
| 300 | 0.26 | 0.74 | 0.92 |
| 301 | 0.81 | 0.19 | 0.09 |
| 302 | 0.85 | 0.15 | 0.05 |
| 303 | 0.76 | 0.24 | 0.21 |
| 304 | 0.98 | 0.02 | 0.03 |
| 305 | 0.93 | 0.07 | 0.03 |
| 306 | 0.98 | 0.02 | 0.02 |
| 307 | 0.95 | 0.05 | 0.01 |
| 308 | 0.58 | 0.42 | 0.16 |
| 309 | 0.98 | 0.02 | 0.17 |
| 310 | 0.59 | 0.41 | 0.67 |
| 311 | 0.91 | 0.09 | 0.20 |
| 312 | 0.99 | 0.01 | 0.00 |
| 313 | 0.97 | 0.03 | 0.05 |
| 314 | 0.75 | 0.25 | 0.26 |
| 315 | 0.98 | 0.02 | 0.01 |
| 316 | 1.00 | 0.00 | 0.00 |
| 317 | 0.39 | 0.61 | 0.79 |
| 318 | 0.97 | 0.03 | 0.00 |
| 319 | 0.28 | 0.72 | 0.29 |
| 320 | 0.98 | 0.02 | 0.00 |
| 321 | 0.87 | 0.13 | 0.12 |
| 322 | 0.88 | 0.12 | 0.17 |
| 323 | 0.10 | 0.90 | 0.94 |
| 324 | 0.98 | 0.02 | 0.03 |
| 325 | 0.99 | 0.01 | 0.00 |
| 326 | 0.99 | 0.01 | 0.04 |
| 327 | 0.89 | 0.11 | 0.10 |
| 328 | 0.50 | 0.50 | 0.39 |
| 329 | 0.88 | 0.12 | 0.08 |
| 330 | 0.71 | 0.29 | 0.39 |
| 331 | 0.61 | 0.39 | 0.17 |
| 332 | 0.96 | 0.04 | 0.07 |
| 333 | 0.96 | 0.04 | 0.05 |
| 334 | 0.63 | 0.37 | 0.33 |
| 335 | 0.62 | 0.38 | 0.15 |
| 336 | 0.97 | 0.03 | 0.01 |
| 337 | 0.57 | 0.43 | 0.50 |
| 338 | 0.90 | 0.10 | 0.07 |
| 339 | 1.00 | 0.00 | 0.02 |
| 340 | 0.73 | 0.27 | 0.35 |
| 341 | 0.69 | 0.31 | 0.04 |
| 342 | 0.99 | 0.01 | 0.01 |
| 343 | 0.98 | 0.02 | 0.06 |
| 344 | 0.94 | 0.06 | 0.08 |
| 345 | 0.88 | 0.12 | 0.03 |
| 346 | 0.98 | 0.02 | 0.04 |
| 347 | 0.91 | 0.09 | 0.26 |
| 348 | 0.99 | 0.01 | 0.00 |
| 349 | 0.78 | 0.22 | 0.19 |
| 350 | 0.94 | 0.06 | 0.04 |
| 351 | 0.88 | 0.12 | 0.10 |
| 352 | 0.96 | 0.04 | 0.01 |
| 353 | 0.99 | 0.01 | 0.01 |
| 354 | 0.99 | 0.01 | 0.01 |
| 355 | 0.31 | 0.69 | 0.51 |
| 356 | 0.68 | 0.32 | 0.40 |
| 357 | 0.98 | 0.02 | 0.13 |
| 358 | 0.63 | 0.37 | 0.23 |
| 359 | 0.99 | 0.01 | 0.01 |
| 360 | 1.00 | 0.00 | 0.02 |
| 361 | 0.66 | 0.34 | 0.95 |
| 362 | 0.98 | 0.02 | 0.00 |
| 363 | 0.99 | 0.01 | 0.02 |
| 364 | 0.99 | 0.01 | 0.02 |
| 365 | 0.99 | 0.01 | 0.02 |
| 366 | 1.00 | 0.00 | 0.03 |
| 367 | 0.99 | 0.01 | 0.02 |
| 368 | 0.99 | 0.01 | 0.03 |
| 369 | 0.99 | 0.01 | 0.01 |
| 370 | 1.00 | 0.00 | 0.02 |
| 371 | 0.99 | 0.01 | 0.00 |
| 372 | 0.98 | 0.02 | 0.01 |
| 373 | 0.75 | 0.25 | 0.16 |
| 374 | 0.81 | 0.19 | 0.17 |
| 375 | 0.96 | 0.04 | 0.49 |
| 376 | 0.98 | 0.02 | 0.04 |
| 377 | 0.44 | 0.56 | 0.39 |
| 378 | 1.00 | 0.00 | 0.02 |
| 379 | 0.99 | 0.01 | 0.03 |
| 380 | 0.95 | 0.05 | 0.22 |
| 381 | 0.99 | 0.01 | 0.00 |
| 382 | 0.99 | 0.01 | 0.02 |
| 383 | 0.98 | 0.02 | 0.01 |
| 384 | 0.48 | 0.52 | 0.71 |
| 385 | 0.85 | 0.15 | 0.15 |
| 386 | 0.97 | 0.03 | 0.08 |
| 387 | 0.42 | 0.58 | 0.82 |
| 388 | 0.74 | 0.26 | 0.14 |
| 389 | 1.00 | 0.00 | 0.03 |
| 390 | 1.00 | 0.00 | 0.01 |
| 391 | 1.00 | 0.00 | 0.00 |
| 392 | 0.57 | 0.43 | 0.02 |
| 393 | 0.99 | 0.01 | 0.04 |
| 394 | 0.74 | 0.26 | 0.31 |
| 395 | 0.78 | 0.22 | 0.58 |
| 396 | 0.92 | 0.08 | 0.02 |
| 397 | 0.97 | 0.03 | 0.13 |
| 398 | 0.92 | 0.08 | 0.07 |
| 399 | 1.00 | 0.00 | 0.02 |
| 400 | 0.89 | 0.11 | 0.07 |
| 401 | 0.98 | 0.02 | 0.06 |
| 402 | 0.98 | 0.02 | 0.09 |
| 403 | 0.78 | 0.22 | 0.25 |
| 404 | 0.97 | 0.03 | 0.09 |
| 405 | 0.98 | 0.02 | 0.02 |
| 406 | 0.99 | 0.01 | 0.04 |
| 407 | 0.88 | 0.12 | 0.18 |
| 408 | 0.85 | 0.15 | 0.05 |
| 409 | 0.36 | 0.64 | 0.49 |
| 410 | 0.99 | 0.01 | 0.01 |
| 411 | 1.00 | 0.00 | 0.01 |
| 412 | 0.93 | 0.07 | 0.01 |
| 413 | 0.98 | 0.02 | 0.02 |
| 414 | 0.97 | 0.03 | 0.01 |
| 415 | 0.87 | 0.13 | 0.04 |
| 416 | 0.56 | 0.44 | 0.18 |
| 417 | 0.98 | 0.02 | 0.00 |
| 418 | 0.88 | 0.12 | 0.26 |
| 419 | 0.86 | 0.14 | 0.10 |
| 420 | 0.94 | 0.06 | 0.02 |
| 421 | 0.99 | 0.01 | 0.05 |
| 422 | 0.61 | 0.39 | 0.32 |
| 423 | 0.89 | 0.11 | 0.08 |
| 424 | 0.91 | 0.09 | 0.08 |
| 425 | 0.80 | 0.20 | 0.26 |
| 426 | 0.99 | 0.01 | 0.02 |
| 427 | 1.00 | 0.00 | 0.01 |
| 428 | 1.00 | 0.00 | 0.02 |
| 429 | 0.37 | 0.63 | 0.55 |
| 430 | 0.60 | 0.40 | 0.22 |
| 431 | 0.95 | 0.05 | 0.07 |
| 432 | 0.90 | 0.10 | 0.69 |
| 433 | 0.93 | 0.07 | 0.11 |
| 434 | 0.98 | 0.02 | 0.02 |
| 435 | 0.79 | 0.21 | 0.15 |
| 436 | 0.92 | 0.08 | 0.15 |
| 437 | 1.00 | 0.00 | 0.01 |
| 438 | 0.98 | 0.02 | 0.00 |
| 439 | 0.99 | 0.01 | 0.01 |
| 440 | 1.00 | 0.00 | 0.03 |
| 441 | 0.48 | 0.52 | 0.13 |
| 442 | 0.97 | 0.03 | 0.11 |
| 443 | 0.99 | 0.01 | 0.05 |
| 444 | 0.66 | 0.34 | 0.17 |
| 445 | 1.00 | 0.00 | 0.02 |
| 446 | 1.00 | 0.00 | 0.02 |
| 447 | 0.57 | 0.43 | 0.54 |
| 448 | 0.97 | 0.03 | 0.11 |
| 449 | 0.77 | 0.23 | 0.22 |
| 450 | 1.00 | 0.00 | 0.03 |
| 451 | 0.73 | 0.27 | 0.18 |
| 452 | 0.99 | 0.01 | 0.01 |
| 453 | 0.99 | 0.01 | 0.02 |
| 454 | 1.00 | 0.00 | 0.01 |
| 455 | 1.00 | 0.00 | 0.02 |
| 456 | 0.98 | 0.02 | 0.02 |
| 457 | 0.99 | 0.01 | 0.01 |
| 458 | 0.99 | 0.01 | 0.02 |
| 459 | 0.99 | 0.01 | 0.02 |
| 460 | 1.00 | 0.00 | 0.03 |
| 461 | 0.83 | 0.17 | 0.91 |
| 462 | 0.97 | 0.03 | 0.02 |
| 463 | 1.00 | 0.00 | 0.01 |
| 464 | 0.96 | 0.04 | 0.01 |
| 465 | 0.99 | 0.01 | 0.00 |
| 466 | 0.95 | 0.05 | 0.44 |
| 467 | 0.97 | 0.03 | 0.04 |
| 468 | 0.99 | 0.01 | 0.02 |
| 469 | 0.16 | 0.84 | 0.87 |
| 470 | 1.00 | 0.00 | 0.20 |
| 471 | 0.68 | 0.32 | 0.06 |
| 472 | 0.53 | 0.47 | 0.96 |
| 473 | 1.00 | 0.00 | 0.01 |
| 474 | 0.94 | 0.06 | 0.02 |
| 475 | 0.50 | 0.50 | 0.52 |
| 476 | 0.97 | 0.03 | 0.05 |
| 477 | 0.97 | 0.03 | 0.00 |
| 478 | 0.99 | 0.01 | 0.01 |
| 479 | 0.94 | 0.06 | 0.06 |
| 480 | 0.20 | 0.80 | 1.06 |
| 481 | 0.98 | 0.02 | 0.01 |
| 482 | 0.93 | 0.07 | 0.11 |
| 483 | 0.51 | 0.49 | 0.85 |
| 484 | 0.97 | 0.03 | 0.06 |
| 485 | 0.96 | 0.04 | 0.02 |
| 486 | 0.58 | 0.42 | 0.17 |
| 487 | 1.00 | 0.00 | 0.01 |
| 488 | 1.00 | 0.00 | 0.03 |
| 489 | 0.98 | 0.02 | 0.06 |
| 490 | 0.85 | 0.15 | 0.30 |
| 491 | 1.00 | 0.00 | 0.01 |

**Legend:**

- Patient ID: Unique identifier for each patient in the database
- Predictive Probability: The calculated probability using LIME analysis for understanding model predictions
- $\left| \boldsymbol{f(x)} \right|$: The calculated predictive probability using SHAP analysis for understanding model predictions
